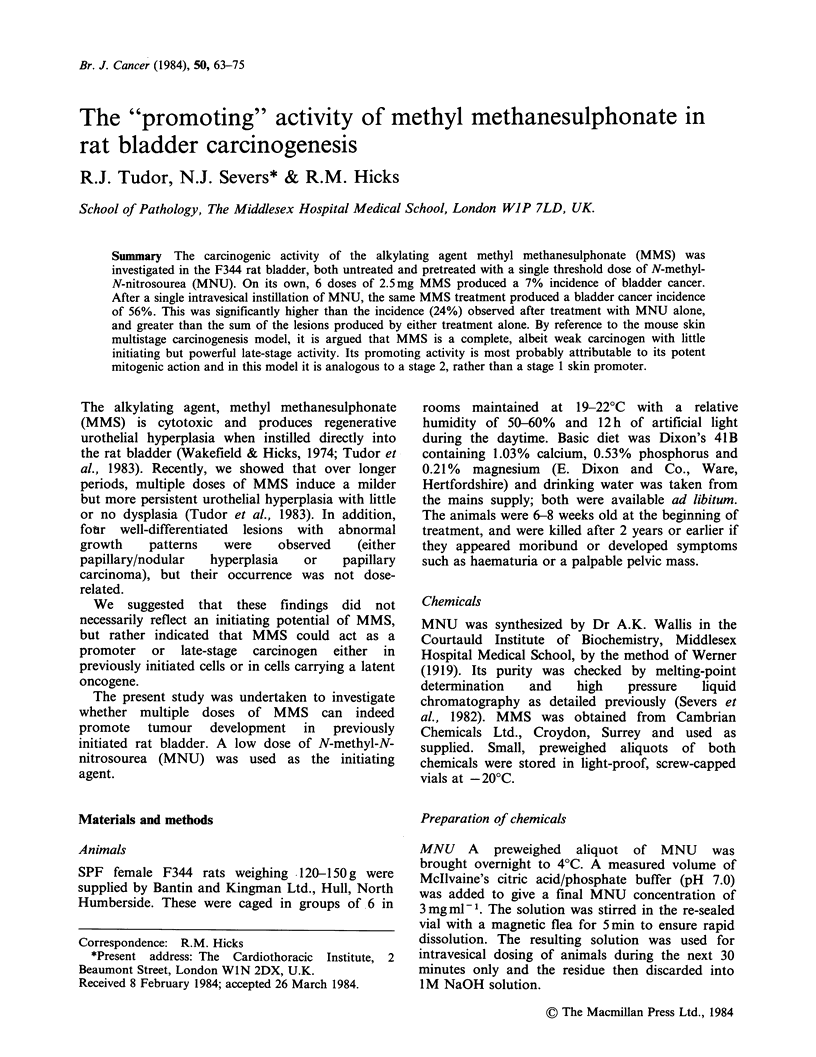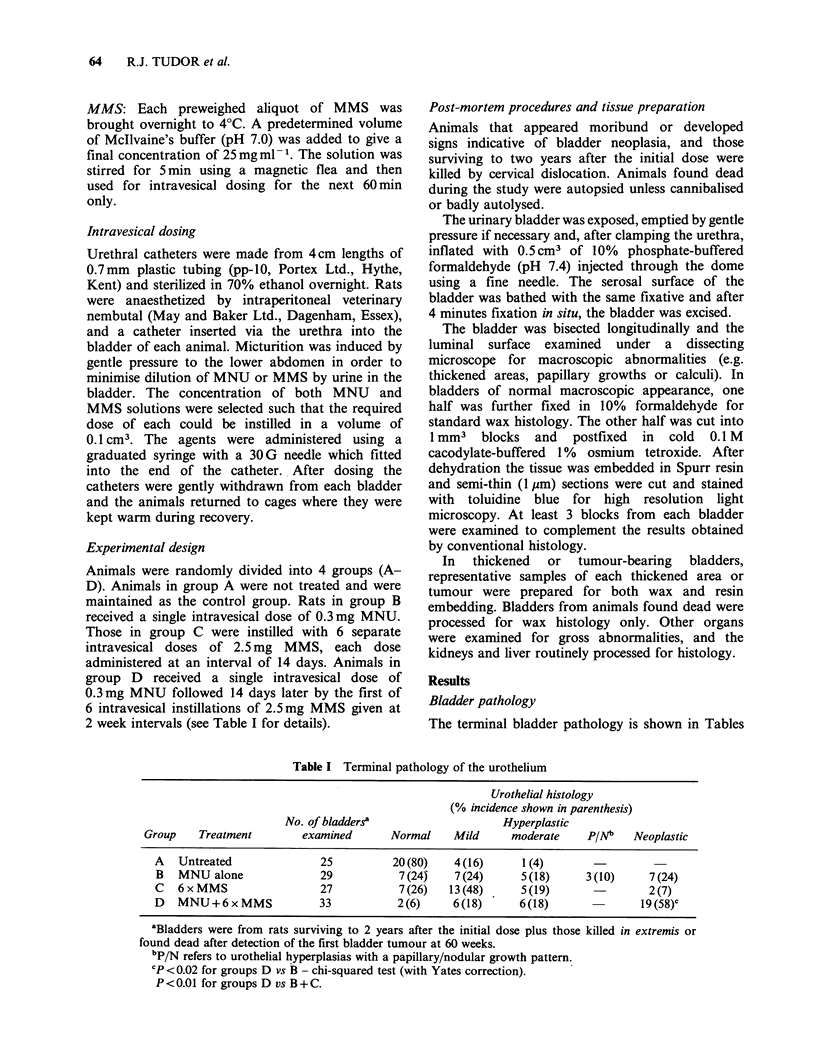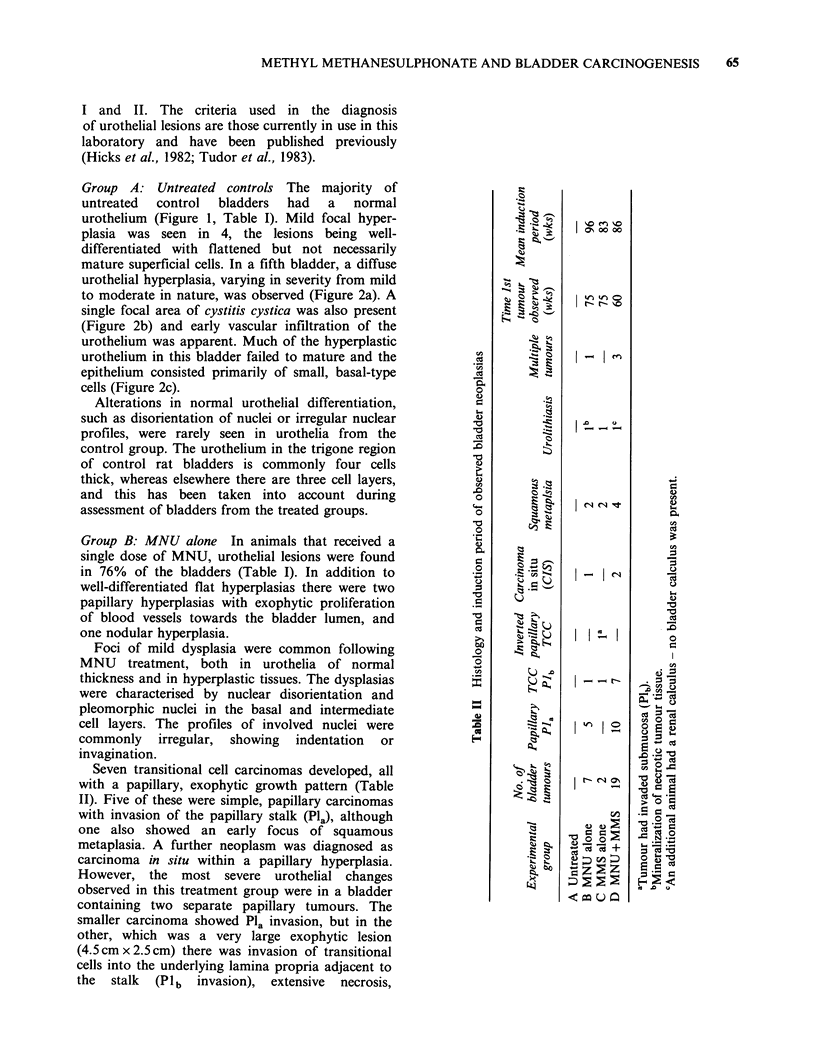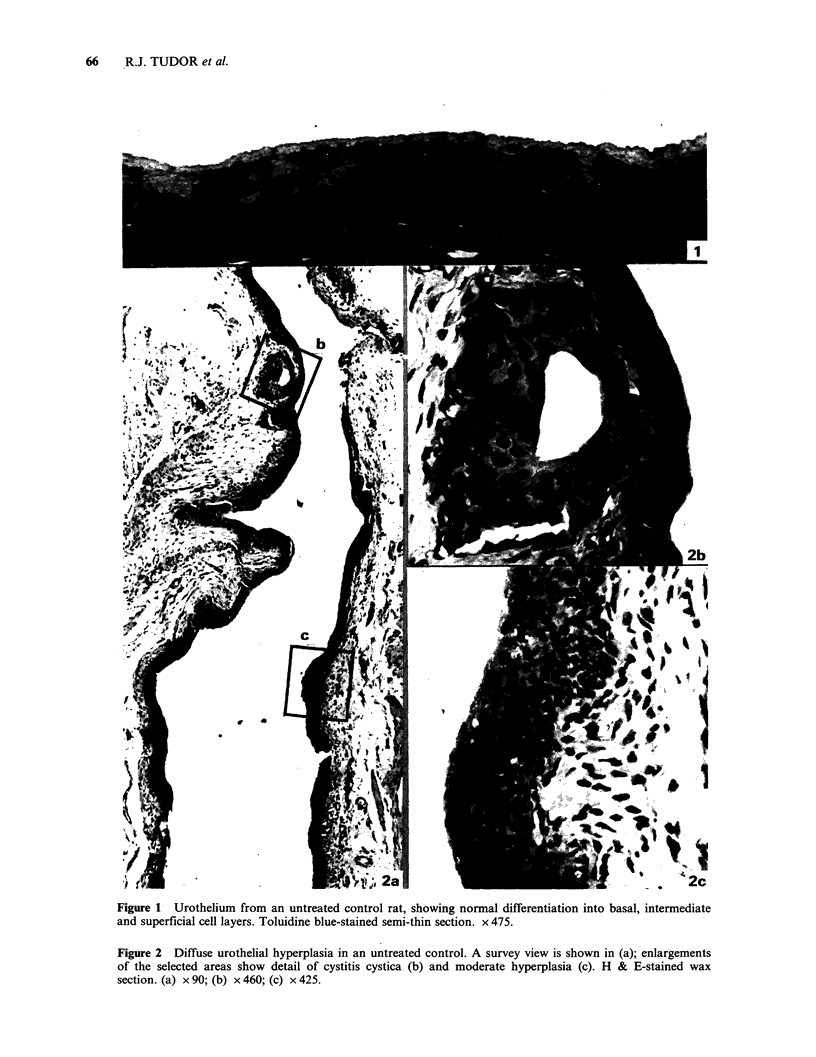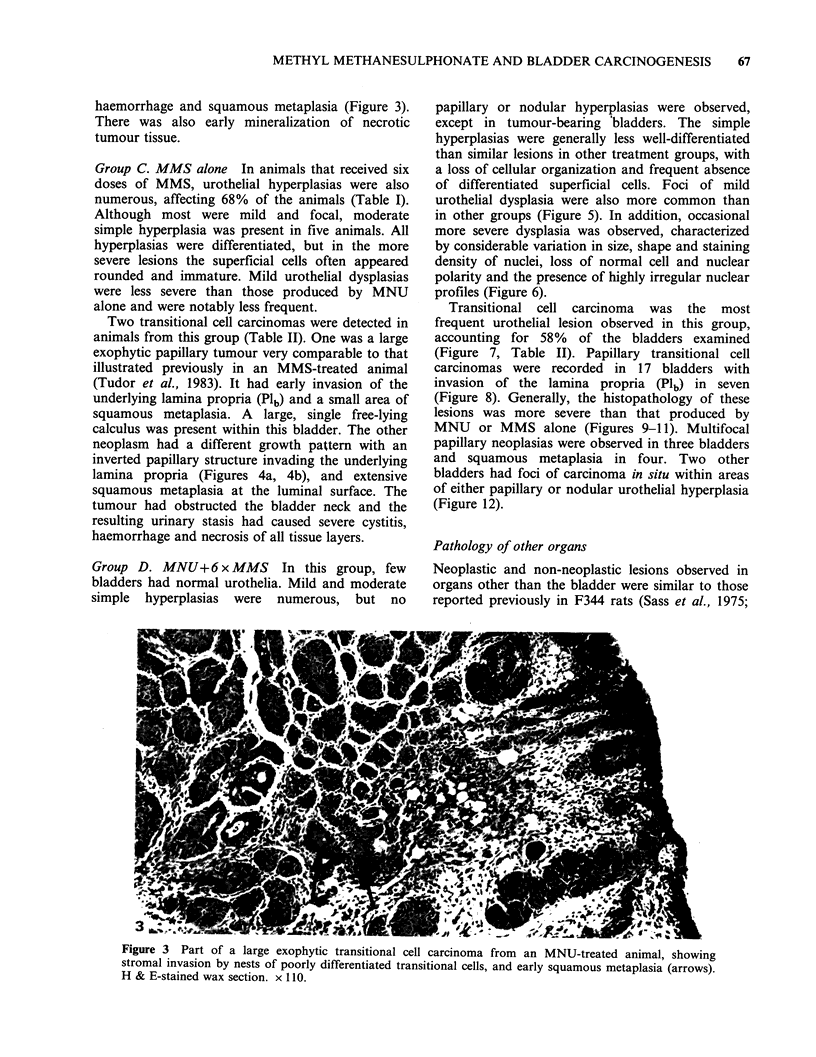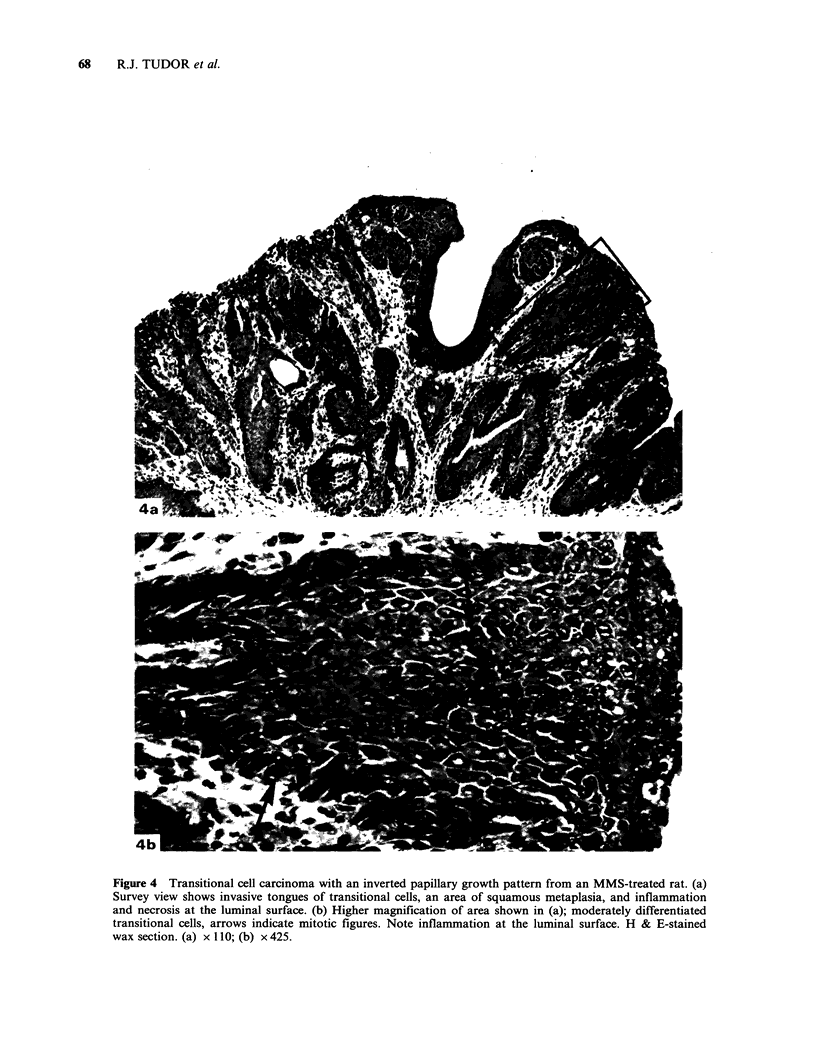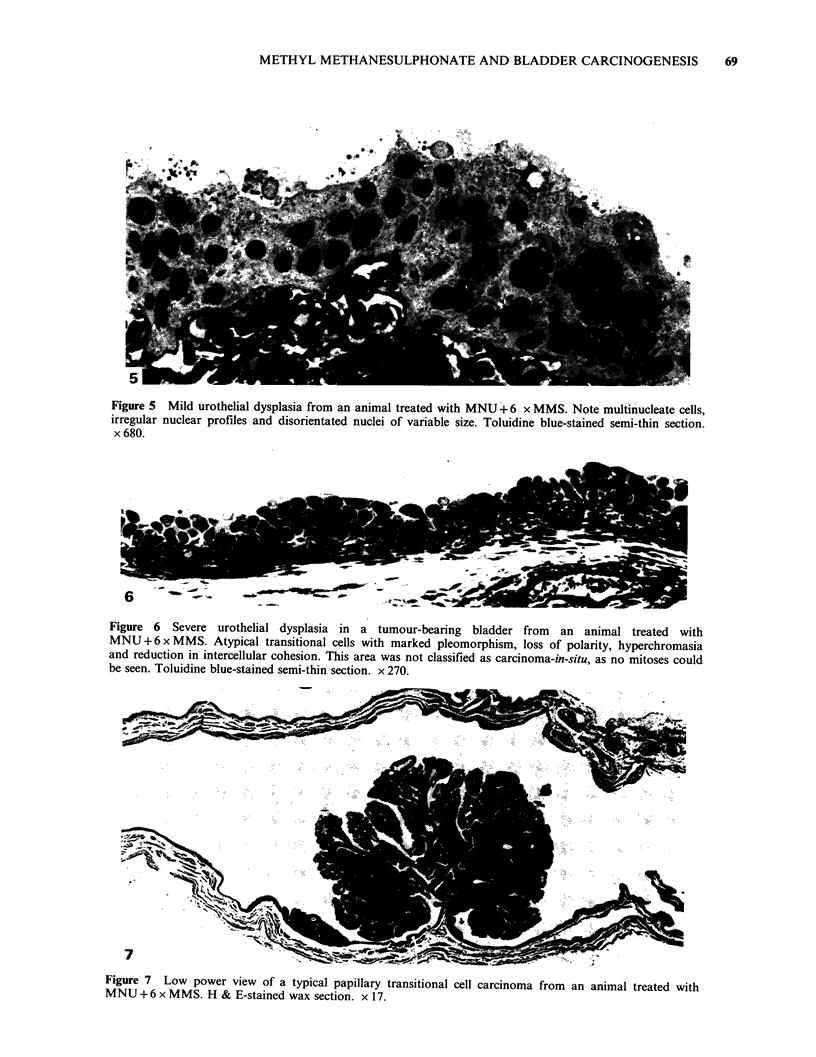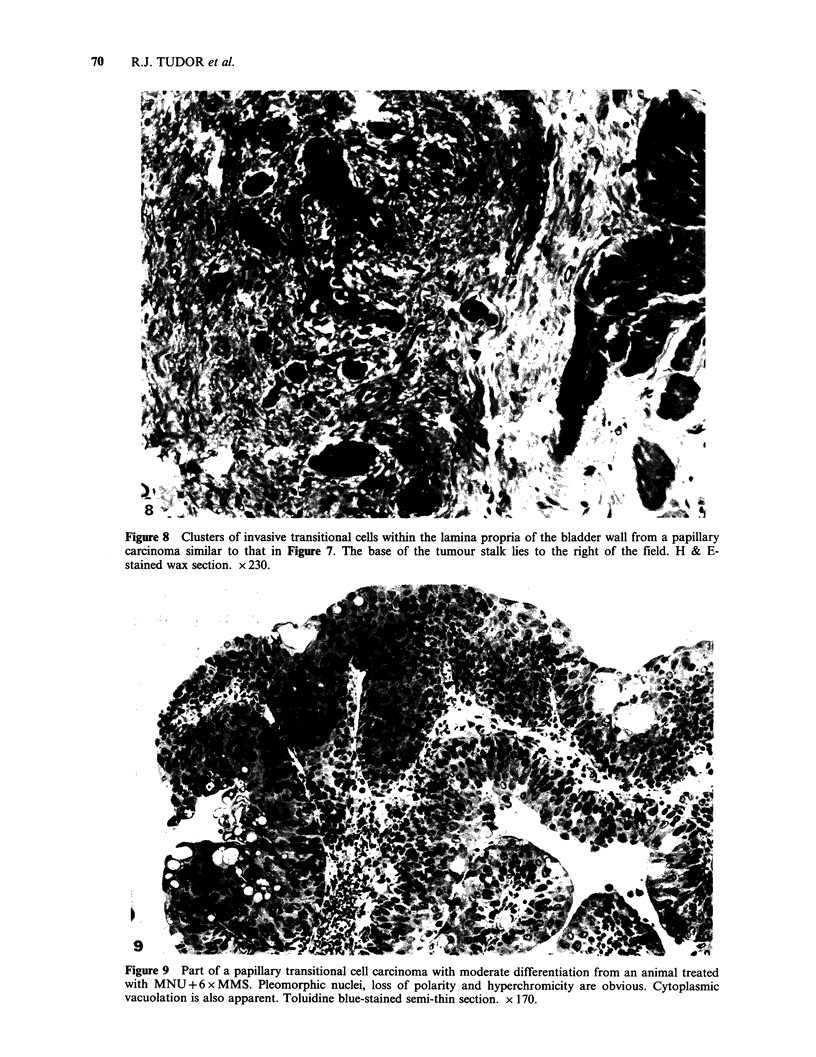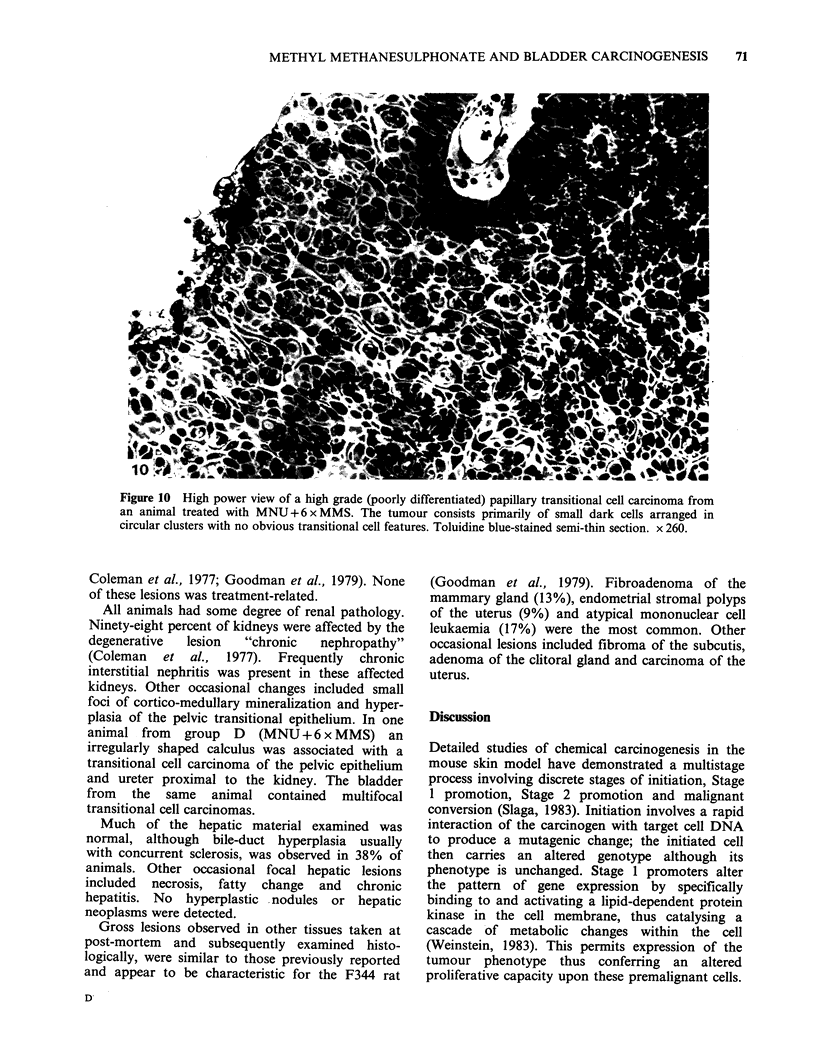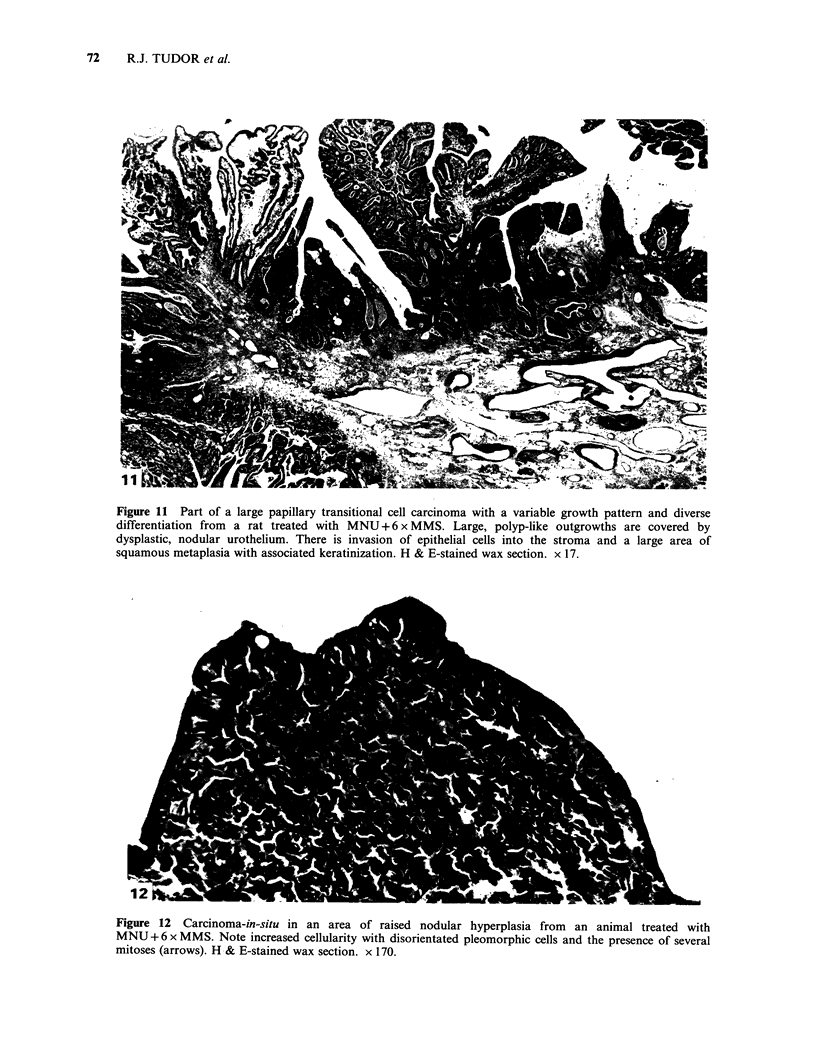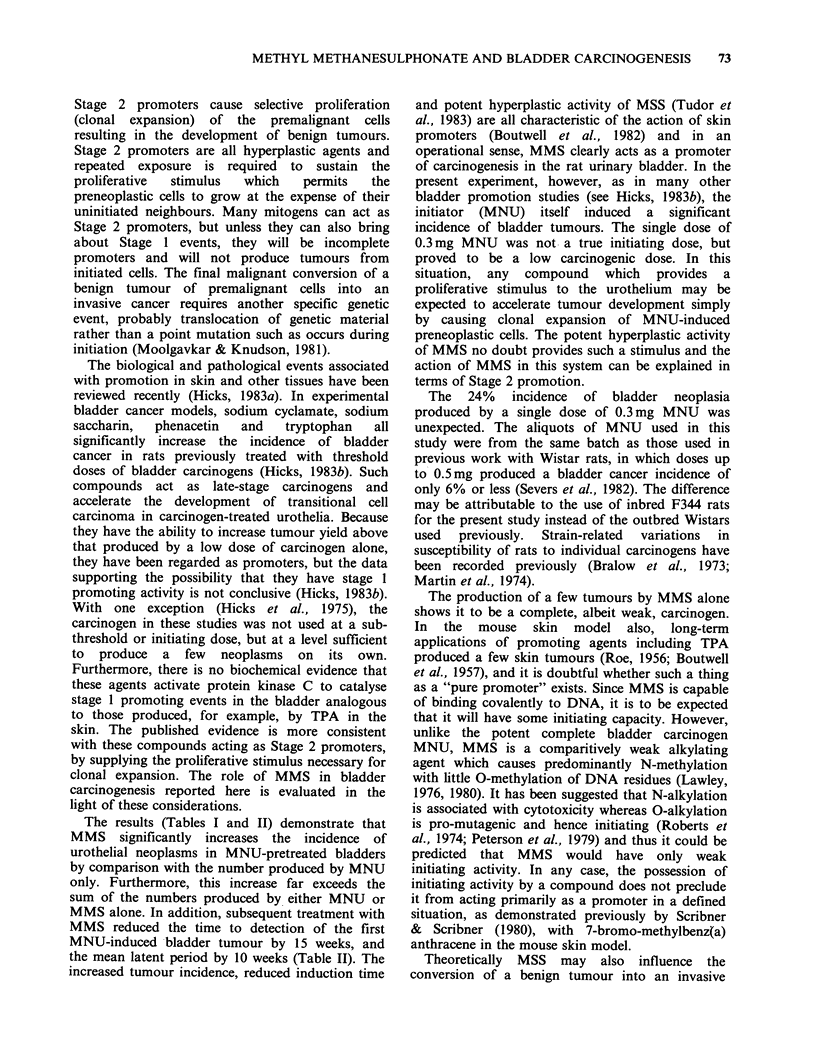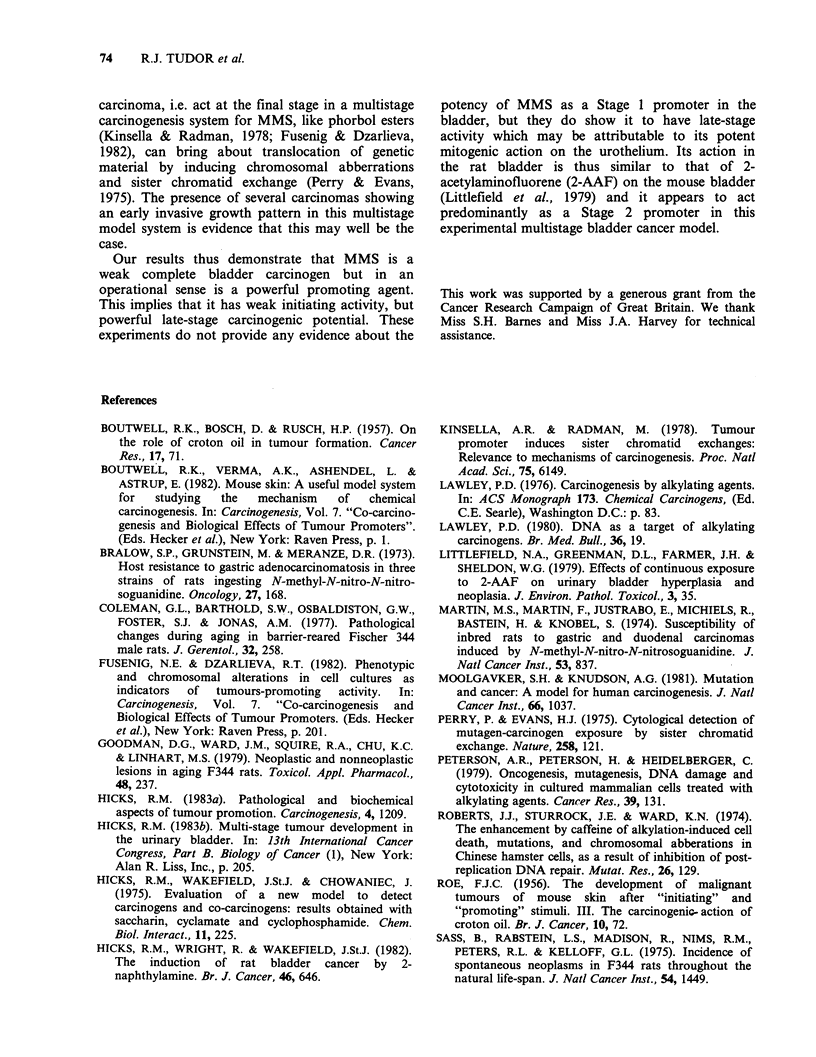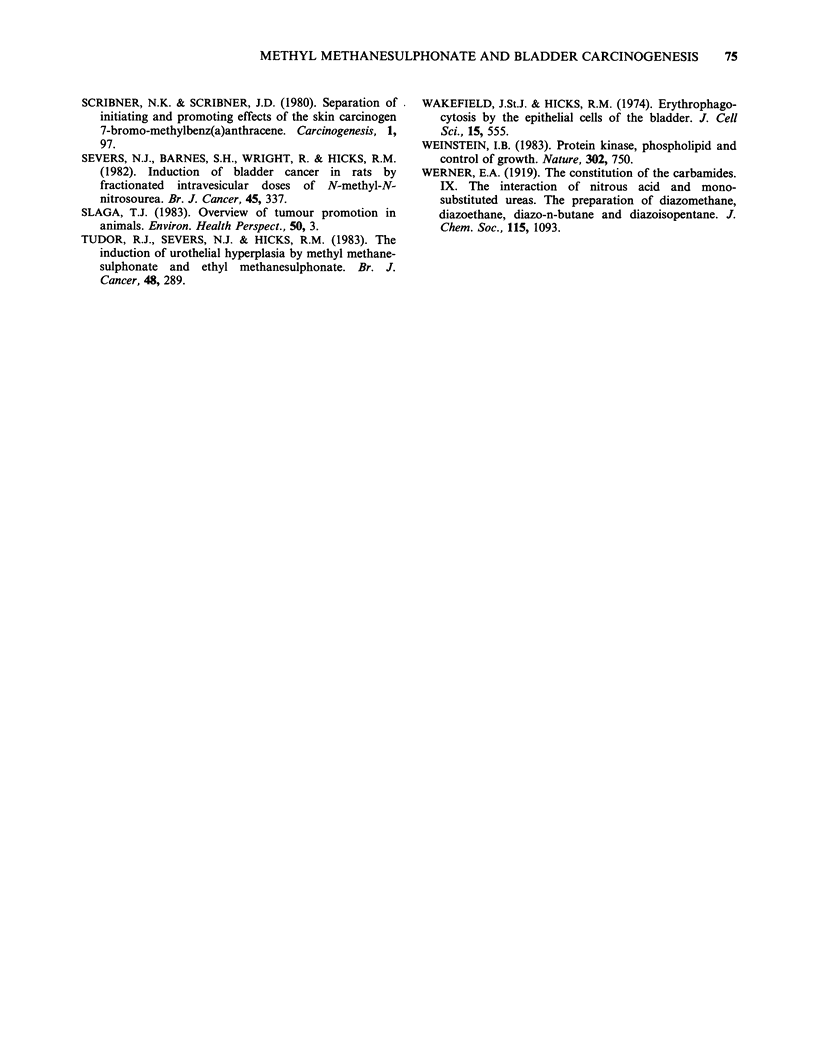# The "promoting" activity of methyl methanesulphonate in rat bladder carcinogenesis.

**DOI:** 10.1038/bjc.1984.140

**Published:** 1984-07

**Authors:** R. J. Tudor, N. J. Severs, R. M. Hicks

## Abstract

**Images:**


*Br. J. Cancer* (1984), **50**, 63–75

### The "promoting" activity of methyl methanesulphonate in rat bladder carcinogenesis

R.J. Tudor, N.J. Severs* & R.M. Hicks

*School of Pathology, The Middlesex Hospital Medical School, London W1P 7LD, UK.*

**Summary** The carcinogenic activity of the alkylating agent methyl methanesulphonate (MMS) was investigated in the F344 rat bladder, both untreated and pretreated with a single threshold dose of *N*-methyl-*N*-nitrosourea (MNU). On its own, 6 doses of 2.5 mg MMS produced a 7% incidence of bladder cancer. After a single intravesical instillation of MNU, the same MMS treatment produced a bladder cancer incidence of 56%. This was significantly higher than the incidence (24%) observed after treatment with MNU alone, and greater than the sum of the lesions produced by either treatment alone. By reference to the mouse skin multistage carcinogenesis model, it is argued that MMS is a complete, albeit weak carcinogen with little initiating but powerful late-stage activity. Its promoting activity is most probably attributable to its potent mitogenic action and in this model it is analogous to a stage 2, rather than a stage 1 skin promoter.

The alkylating agent, methyl methanesulphonate (MMS) is cytotoxic and produces regenerative urothelial hyperplasia when instilled directly into the rat bladder (Wakefield & Hicks, 1974; Tudor *et al.*, 1983). Recently, we showed that over longer periods, multiple doses of MMS induce a milder but more persistent urothelial hyperplasia with little or no dysplasia (Tudor *et al.*, 1983). In addition, four well-differentiated lesions with abnormal growth patterns were observed (either papillary/nodular hyperplasia or papillary carcinoma), but their occurrence was not dose-related.

We suggested that these findings did not necessarily reflect an initiating potential of MMS, but rather indicated that MMS could act as a promoter or late-stage carcinogen either in previously initiated cells or in cells carrying a latent oncogene.

The present study was undertaken to investigate whether multiple doses of MMS can indeed promote tumour development in previously initiated rat bladder. A low dose of *N*-methyl-*N*-nitrosourea (MNU) was used as the initiating agent.

### Materials and methods

### *Animals*

SPF female F344 rats weighing 120–150 g were supplied by Bantin and Kingman Ltd., Hull, North Humberside. These were caged in groups of 6 in rooms maintained at 19–22°C with a relative humidity of 50–60% and 12 h of artificial light during the daytime. Basic diet was Dixon's 41B containing 1.03% calcium, 0.53% phosphorus and 0.21% magnesium (E. Dixon and Co., Ware, Hertfordshire) and drinking water was taken from the mains supply; both were available *ad libitum*. The animals were 6–8 weeks old at the beginning of treatment, and were killed after 2 years or earlier if they appeared moribund or developed symptoms such as haematuria or a palpable pelvic mass.

### *Chemicals*

MNU was synthesized by Dr A.K. Wallis in the Courtauld Institute of Biochemistry, Middlesex Hospital Medical School, by the method of Werner (1919). Its purity was checked by melting-point determination and high pressure liquid chromatography as detailed previously (Severs *et al.*, 1982). MMS was obtained from Cambrian Chemicals Ltd., Croydon, Surrey and used as supplied. Small, preweighed aliquots of both chemicals were stored in light-proof, screw-capped vials at −20°C.

### *Preparation of chemicals*

*MNU* A preweighed aliquot of MNU was brought overnight to 4°C. A measured volume of McIlvaine's citric acid/phosphate buffer (pH 7.0) was added to give a final MNU concentration of 3 mg ml$^{-1}$. The solution was stirred in the re-sealed vial with a magnetic flea for 5 min to ensure rapid dissolution. The resulting solution was used for intravesical dosing of animals during the next 30 minutes only and the residue then discarded into 1M NaOH solution.

Correspondence: R.M. Hicks

*Present address: The Cardiothoracic Institute, 2 Beaumont Street, London W1N 2DX, U.K.

Received 8 February 1984; accepted 26 March 1984.

© The Macmillan Press Ltd., 1984

*MMS*: Each preweighed aliquot of MMS was brought overnight to 4°C. A predetermined volume of McIlvaine's buffer (pH 7.0) was added to give a final concentration of $25\,mg\,ml^{-1}$. The solution was stirred for 5 min using a magnetic flea and then used for intravesical dosing for the next 60 min only.

*Intravesical dosing*

Urethral catheters were made from 4 cm lengths of 0.7 mm plastic tubing (pp-10, Portex Ltd., Hythe, Kent) and sterilized in 70% ethanol overnight. Rats were anaesthetized by intraperitoneal veterinary nembutal (May and Baker Ltd., Dagenham, Essex), and a catheter inserted via the urethra into the bladder of each animal. Micturition was induced by gentle pressure to the lower abdomen in order to minimise dilution of MNU or MMS by urine in the bladder. The concentration of both MNU and MMS solutions were selected such that the required dose of each could be instilled in a volume of $0.1\,cm^3$. The agents were administered using a graduated syringe with a 30 G needle which fitted into the end of the catheter. After dosing the catheters were gently withdrawn from each bladder and the animals returned to cages where they were kept warm during recovery.

*Experimental design*

Animals were randomly divided into 4 groups (A–D). Animals in group A were not treated and were maintained as the control group. Rats in group B received a single intravesical dose of 0.3 mg MNU. Those in group C were instilled with 6 separate intravesical doses of 2.5 mg MMS, each dose administered at an interval of 14 days. Animals in group D received a single intravesical dose of 0.3 mg MNU followed 14 days later by the first of 6 intravesical instillations of 2.5 mg MMS given at 2 week intervals (see Table I for details).

*Post-mortem procedures and tissue preparation*

Animals that appeared moribund or developed signs indicative of bladder neoplasia, and those surviving to two years after the initial dose were killed by cervical dislocation. Animals found dead during the study were autopsied unless cannibalised or badly autolysed.

The urinary bladder was exposed, emptied by gentle pressure if necessary and, after clamping the urethra, inflated with $0.5\,cm^3$ of 10% phosphate-buffered formaldehyde (pH 7.4) injected through the dome using a fine needle. The serosal surface of the bladder was bathed with the same fixative and after 4 minutes fixation *in situ*, the bladder was excised.

The bladder was bisected longitudinally and the luminal surface examined under a dissecting microscope for macroscopic abnormalities (e.g. thickened areas, papillary growths or calculi). In bladders of normal macroscopic appearance, one half was further fixed in 10% formaldehyde for standard wax histology. The other half was cut into $1\,mm^3$ blocks and postfixed in cold 0.1 M cacodylate-buffered 1% osmium tetroxide. After dehydration the tissue was embedded in Spurr resin and semi-thin (1 $\mu$m) sections were cut and stained with toluidine blue for high resolution light microscopy. At least 3 blocks from each bladder were examined to complement the results obtained by conventional histology.

In thickened or tumour-bearing bladders, representative samples of each thickened area or tumour were prepared for both wax and resin embedding. Bladders from animals found dead were processed for wax histology only. Other organs were examined for gross abnormalities, and the kidneys and liver routinely processed for histology.

### Results

*Bladder pathology*

The terminal bladder pathology is shown in Tables

**Table I** Terminal pathology of the urothelium

| | | | *Urothelial histology (% incidence shown in parenthesis)* | | | | |
|---|---|---|---|---|---|---|---|
| | | | | | *Hyperplastic* | | |
| *Group* | *Treatment* | *No. of bladders*[a] *examined* | *Normal* | *Mild* | *moderate* | *P/N*[b] | *Neoplastic* |
| A | Untreated | 25 | 20 (80) | 4 (16) | 1 (4) | — | — |
| B | MNU alone | 29 | 7 (24) | 7 (24) | 5 (18) | 3 (10) | 7 (24) |
| C | 6 × MMS | 27 | 7 (26) | 13 (48) | 5 (19) | — | 2 (7) |
| D | MNU + 6 × MMS | 33 | 2 (6) | 6 (18) | 6 (18) | — | 19 (58)[c] |

[a]Bladders were from rats surviving to 2 years after the initial dose plus those killed *in extremis* or found dead after detection of the first bladder tumour at 60 weeks.
[b]P/N refers to urothelial hyperplasias with a papillary/nodular growth pattern.
[c]$P < 0.02$ for groups D *vs* B – chi-squared test (with Yates correction).
$P < 0.01$ for groups D *vs* B + C.

I and II. The criteria used in the diagnosis of urothelial lesions are those currently in use in this laboratory and have been published previously (Hicks *et al.*, 1982; Tudor *et al.*, 1983).

*Group A: Untreated controls* The majority of untreated control bladders had a normal urothelium (Figure 1, Table I). Mild focal hyperplasia was seen in 4, the lesions being well-differentiated with flattened but not necessarily mature superficial cells. In a fifth bladder, a diffuse urothelial hyperplasia, varying in severity from mild to moderate in nature, was observed (Figure 2a). A single focal area of *cystitis cystica* was also present (Figure 2b) and early vascular infiltration of the urothelium was apparent. Much of the hyperplastic urothelium in this bladder failed to mature and the epithelium consisted primarily of small, basal-type cells (Figure 2c).

Alterations in normal urothelial differentiation, such as disorientation of nuclei or irregular nuclear profiles, were rarely seen in urothelia from the control group. The urothelium in the trigone region of control rat bladders is commonly four cells thick, whereas elsewhere there are three cell layers, and this has been taken into account during assessment of bladders from the treated groups.

*Group B: MNU alone* In animals that received a single dose of MNU, urothelial lesions were found in 76% of the bladders (Table I). In addition to well-differentiated flat hyperplasias there were two papillary hyperplasias with exophytic proliferation of blood vessels towards the bladder lumen, and one nodular hyperplasia.

Foci of mild dysplasia were common following MNU treatment, both in urothelia of normal thickness and in hyperplastic tissues. The dysplasias were characterised by nuclear disorientation and pleomorphic nuclei in the basal and intermediate cell layers. The profiles of involved nuclei were commonly irregular, showing indentation or invagination.

Seven transitional cell carcinomas developed, all with a papillary, exophytic growth pattern (Table II). Five of these were simple, papillary carcinomas with invasion of the papillary stalk ($Pl_a$), although one also showed an early focus of squamous metaplasia. A further neoplasm was diagnosed as carcinoma *in situ* within a papillary hyperplasia. However, the most severe urothelial changes observed in this treatment group were in a bladder containing two separate papillary tumours. The smaller carcinoma showed $Pl_a$ invasion, but in the other, which was a very large exophytic lesion (4.5 cm x 2.5 cm) there was invasion of transitional cells into the underlying lamina propria adjacent to the stalk ($Pl_b$ invasion), extensive necrosis,

**Table II** Histology and induction period of observed bladder neoplasias

| Experimental group | No. of bladder tumours | Papillary $Pl_a$ | TCC $Pl_b$ | Inverted papillary TCC | Carcinoma in situ (CIS) | Squamous metaplsia | Urolithiasis | Multiple tumours | Time 1st tumour observed (wks) | Mean induction period (wks) |
|---|---|---|---|---|---|---|---|---|---|---|
| A Untreated | — | — | — | — | — | — | — | — | — | — |
| B MNU alone | 7 | 5 | 1 | — | 1 | 2 | 1[b] | 1 | 75 | 96 |
| C MMS alone | 2 | — | 1 | 1[a] | — | 2 | 1 | — | 75 | 83 |
| D MNU+MMS | 19 | 10 | 7 | — | 2 | 4 | 1[c] | 3 | 60 | 86 |

[a]Tumour had invaded submucosa ($Pl_b$).

[b]Mineralization of necrotic tumour tissue.

[c]An additional animal had a renal calculus – no bladder calculus was present.

**Figure 1** Urothelium from an untreated control rat, showing normal differentiation into basal, intermediate and superficial cell layers. Toluidine blue-stained semi-thin section. ×475.

**Figure 2** Diffuse urothelial hyperplasia in an untreated control. A survey view is shown in (a); enlargements of the selected areas show detail of cystitis cystica (b) and moderate hyperplasia (c). H & E-stained wax section. (a) ×90; (b) ×460; (c) ×425.

haemorrhage and squamous metaplasia (Figure 3). There was also early mineralization of necrotic tumour tissue.

*Group C. MMS alone* In animals that received six doses of MMS, urothelial hyperplasias were also numerous, affecting 68% of the animals (Table I). Although most were mild and focal, moderate simple hyperplasia was present in five animals. All hyperplasias were differentiated, but in the more severe lesions the superficial cells often appeared rounded and immature. Mild urothelial dysplasias were less severe than those produced by MNU alone and were notably less frequent.

Two transitional cell carcinomas were detected in animals from this group (Table II). One was a large exophytic papillary tumour very comparable to that illustrated previously in an MMS-treated animal (Tudor *et al.*, 1983). It had early invasion of the underlying lamina propria ($Pl_b$) and a small area of squamous metaplasia. A large, single free-lying calculus was present within this bladder. The other neoplasm had a different growth pattern with an inverted papillary structure invading the underlying lamina propria (Figures 4a, 4b), and extensive squamous metaplasia at the luminal surface. The tumour had obstructed the bladder neck and the resulting urinary stasis had caused severe cystitis, haemorrhage and necrosis of all tissue layers.

*Group D. MNU+6×MMS* In this group, few bladders had normal urothelia. Mild and moderate simple hyperplasias were numerous, but no papillary or nodular hyperplasias were observed, except in tumour-bearing bladders. The simple hyperplasias were generally less well-differentiated than similar lesions in other treatment groups, with a loss of cellular organization and frequent absence of differentiated superficial cells. Foci of mild urothelial dysplasia were also more common than in other groups (Figure 5). In addition, occasional more severe dysplasia was observed, characterized by considerable variation in size, shape and staining density of nuclei, loss of normal cell and nuclear polarity and the presence of highly irregular nuclear profiles (Figure 6).

Transitional cell carcinoma was the most frequent urothelial lesion observed in this group, accounting for 58% of the bladders examined (Figure 7, Table II). Papillary transitional cell carcinomas were recorded in 17 bladders with invasion of the lamina propria ($Pl_b$) in seven (Figure 8). Generally, the histopathology of these lesions was more severe than that produced by MNU or MMS alone (Figures 9–11). Multifocal papillary neoplasias were observed in three bladders and squamous metaplasia in four. Two other bladders had foci of carcinoma *in situ* within areas of either papillary or nodular urothelial hyperplasia (Figure 12).

### *Pathology of other organs*

Neoplastic and non-neoplastic lesions observed in organs other than the bladder were similar to those reported previously in F344 rats (Sass *et al.*, 1975;

**Figure 3** Part of a large exophytic transitional cell carcinoma from an MNU-treated animal, showing stromal invasion by nests of poorly differentiated transitional cells, and early squamous metaplasia (arrows). H & E-stained wax section. ×110.

**Figure 4** Transitional cell carcinoma with an inverted papillary growth pattern from an MMS-treated rat. (a) Survey view shows invasive tongues of transitional cells, an area of squamous metaplasia, and inflammation and necrosis at the luminal surface. (b) Higher magnification of area shown in (a); moderately differentiated transitional cells, arrows indicate mitotic figures. Note inflammation at the luminal surface. H & E-stained wax section. (a) $\times 110$; (b) $\times 425$.

**Figure 5** Mild urothelial dysplasia from an animal treated with MNU+6 ×MMS. Note multinucleate cells, irregular nuclear profiles and disorientated nuclei of variable size. Toluidine blue-stained semi-thin section. ×680.

**Figure 6** Severe urothelial dysplasia in a tumour-bearing bladder from an animal treated with MNU+6×MMS. Atypical transitional cells with marked pleomorphism, loss of polarity, hyperchromasia and reduction in intercellular cohesion. This area was not classified as carcinoma-*in-situ*, as no mitoses could be seen. Toluidine blue-stained semi-thin section. ×270.

**Figure 7** Low power view of a typical papillary transitional cell carcinoma from an animal treated with MNU+6×MMS. H & E-stained wax section. ×17.

**Figure 8** Clusters of invasive transitional cells within the lamina propria of the bladder wall from a papillary carcinoma similar to that in **Figure** 7. The base of the tumour stalk lies to the right of the field. H & E-stained wax section. ×230.

**Figure 9** Part of a papillary transitional cell carcinoma with moderate differentiation from an animal treated with MNU + 6 × MMS. Pleomorphic nuclei, loss of polarity and hyperchromicity are obvious. Cytoplasmic vacuolation is also apparent. Toluidine blue-stained semi-thin section. ×170.

**Figure 10** High power view of a high grade (poorly differentiated) papillary transitional cell carcinoma from an animal treated with MNU+6×MMS. The tumour consists primarily of small dark cells arranged in circular clusters with no obvious transitional cell features. Toluidine blue-stained semi-thin section. ×260.

Coleman *et al.*, 1977; Goodman *et al.*, 1979). None of these lesions was treatment-related.

All animals had some degree of renal pathology. Ninety-eight percent of kidneys were affected by the degenerative lesion "chronic nephropathy" (Coleman *et al.*, 1977). Frequently chronic interstitial nephritis was present in these affected kidneys. Other occasional changes included small foci of cortico-medullary mineralization and hyperplasia of the pelvic transitional epithelium. In one animal from group D (MNU+6×MMS) an irregularly shaped calculus was associated with a transitional cell carcinoma of the pelvic epithelium and ureter proximal to the kidney. The bladder from the same animal contained multifocal transitional cell carcinomas.

Much of the hepatic material examined was normal, although bile-duct hyperplasia usually with concurrent sclerosis, was observed in 38% of animals. Other occasional focal hepatic lesions included necrosis, fatty change and chronic hepatitis. No hyperplastic nodules or hepatic neoplasms were detected.

Gross lesions observed in other tissues taken at post-mortem and subsequently examined histologically, were similar to those previously reported and appear to be characteristic for the F344 rat (Goodman *et al.*, 1979). Fibroadenoma of the mammary gland (13%), endometrial stromal polyps of the uterus (9%) and atypical mononuclear cell leukaemia (17%) were the most common. Other occasional lesions included fibroma of the subcutis, adenoma of the clitoral gland and carcinoma of the uterus.

### Discussion

Detailed studies of chemical carcinogenesis in the mouse skin model have demonstrated a multistage process involving discrete stages of initiation, Stage 1 promotion, Stage 2 promotion and malignant conversion (Slaga, 1983). Initiation involves a rapid interaction of the carcinogen with target cell DNA to produce a mutagenic change; the initiated cell then carries an altered genotype although its phenotype is unchanged. Stage 1 promoters alter the pattern of gene expression by specifically binding to and activating a lipid-dependent protein kinase in the cell membrane, thus catalysing a cascade of metabolic changes within the cell (Weinstein, 1983). This permits expression of the tumour phenotype thus conferring an altered proliferative capacity upon these premalignant cells.

**Figure 11** Part of a large papillary transitional cell carcinoma with a variable growth pattern and diverse differentiation from a rat treated with MNU + 6 × MMS. Large, polyp-like outgrowths are covered by dysplastic, nodular urothelium. There is invasion of epithelial cells into the stroma and a large area of squamous metaplasia with associated keratinization. H & E-stained wax section. × 17.

**Figure 12** Carcinoma-*in-situ* in an area of raised nodular hyperplasia from an animal treated with MNU + 6 × MMS. Note increased cellularity with disorientated pleomorphic cells and the presence of several mitoses (arrows). H & E-stained wax section. × 170.

Stage 2 promoters cause selective proliferation (clonal expansion) of the premalignant cells resulting in the development of benign tumours. Stage 2 promoters are all hyperplastic agents and repeated exposure is required to sustain the proliferative stimulus which permits the preneoplastic cells to grow at the expense of their uninitiated neighbours. Many mitogens can act as Stage 2 promoters, but unless they can also bring about Stage 1 events, they will be incomplete promoters and will not produce tumours from initiated cells. The final malignant conversion of a benign tumour of premalignant cells into an invasive cancer requires another specific genetic event, probably translocation of genetic material rather than a point mutation such as occurs during initiation (Moolgavkar & Knudson, 1981).

The biological and pathological events associated with promotion in skin and other tissues have been reviewed recently (Hicks, 1983*a*). In experimental bladder cancer models, sodium cyclamate, sodium saccharin, phenacetin and tryptophan all significantly increase the incidence of bladder cancer in rats previously treated with threshold doses of bladder carcinogens (Hicks, 1983*b*). Such compounds act as late-stage carcinogens and accelerate the development of transitional cell carcinoma in carcinogen-treated urothelia. Because they have the ability to increase tumour yield above that produced by a low dose of carcinogen alone, they have been regarded as promoters, but the data supporting the possibility that they have stage 1 promoting activity is not conclusive (Hicks, 1983*b*). With one exception (Hicks *et al.*, 1975), the carcinogen in these studies was not used at a sub-threshold or initiating dose, but at a level sufficient to produce a few neoplasms on its own. Furthermore, there is no biochemical evidence that these agents activate protein kinase C to catalyse stage 1 promoting events in the bladder analogous to those produced, for example, by TPA in the skin. The published evidence is more consistent with these compounds acting as Stage 2 promoters, by supplying the proliferative stimulus necessary for clonal expansion. The role of MMS in bladder carcinogenesis reported here is evaluated in the light of these considerations.

The results (Tables I and II) demonstrate that MMS significantly increases the incidence of urothelial neoplasms in MNU-pretreated bladders by comparison with the number produced by MNU only. Furthermore, this increase far exceeds the sum of the numbers produced by either MNU or MMS alone. In addition, subsequent treatment with MMS reduced the time to detection of the first MNU-induced bladder tumour by 15 weeks, and the mean latent period by 10 weeks (Table II). The increased tumour incidence, reduced induction time and potent hyperplastic activity of MSS (Tudor *et al.*, 1983) are all characteristic of the action of skin promoters (Boutwell *et al.*, 1982) and in an operational sense, MMS clearly acts as a promoter of carcinogenesis in the rat urinary bladder. In the present experiment, however, as in many other bladder promotion studies (see Hicks, 1983*b*), the initiator (MNU) itself induced a significant incidence of bladder tumours. The single dose of 0.3 mg MNU was not a true initiating dose, but proved to be a low carcinogenic dose. In this situation, any compound which provides a proliferative stimulus to the urothelium may be expected to accelerate tumour development simply by causing clonal expansion of MNU-induced preneoplastic cells. The potent hyperplastic activity of MMS no doubt provides such a stimulus and the action of MMS in this system can be explained in terms of Stage 2 promotion.

The 24% incidence of bladder neoplasia produced by a single dose of 0.3 mg MNU was unexpected. The aliquots of MNU used in this study were from the same batch as those used in previous work with Wistar rats, in which doses up to 0.5 mg produced a bladder cancer incidence of only 6% or less (Severs *et al.*, 1982). The difference may be attributable to the use of inbred F344 rats for the present study instead of the outbred Wistars used previously. Strain-related variations in susceptibility of rats to individual carcinogens have been recorded previously (Bralow *et al.*, 1973; Martin *et al.*, 1974).

The production of a few tumours by MMS alone shows it to be a complete, albeit weak, carcinogen. In the mouse skin model also, long-term applications of promoting agents including TPA produced a few skin tumours (Roe, 1956; Boutwell *et al.*, 1957), and it is doubtful whether such a thing as a "pure promoter" exists. Since MMS is capable of binding covalently to DNA, it is to be expected that it will have some initiating capacity. However, unlike the potent complete bladder carcinogen MNU, MMS is a comparitively weak alkylating agent which causes predominantly N-methylation with little O-methylation of DNA residues (Lawley, 1976, 1980). It has been suggested that N-alkylation is associated with cytotoxicity whereas O-alkylation is pro-mutagenic and hence initiating (Roberts *et al.*, 1974; Peterson *et al.*, 1979) and thus it could be predicted that MMS would have only weak initiating activity. In any case, the possession of initiating activity by a compound does not preclude it from acting primarily as a promoter in a defined situation, as demonstrated previously by Scribner & Scribner (1980), with 7-bromo-methylbenz(a) anthracene in the mouse skin model.

Theoretically MSS may also influence the conversion of a benign tumour into an invasive carcinoma, i.e. act at the final stage in a multistage carcinogenesis system for MMS, like phorbol esters (Kinsella & Radman, 1978; Fusenig & Dzarlieva, 1982), can bring about translocation of genetic material by inducing chromosomal abberrations and sister chromatid exchange (Perry & Evans, 1975). The presence of several carcinomas showing an early invasive growth pattern in this multistage model system is evidence that this may well be the case.

Our results thus demonstrate that MMS is a weak complete bladder carcinogen but in an operational sense is a powerful promoting agent. This implies that it has weak initiating activity, but powerful late-stage carcinogenic potential. These experiments do not provide any evidence about the potency of MMS as a Stage 1 promoter in the bladder, but they do show it to have late-stage activity which may be attributable to its potent mitogenic action on the urothelium. Its action in the rat bladder is thus similar to that of 2-acetylaminofluorene (2-AAF) on the mouse bladder (Littlefield *et al.*, 1979) and it appears to act predominantly as a Stage 2 promoter in this experimental multistage bladder cancer model.

This work was supported by a generous grant from the Cancer Research Campaign of Great Britain. We thank Miss S.H. Barnes and Miss J.A. Harvey for technical assistance.

### References

BOUTWELL, R.K., BOSCH, D. & RUSCH, H.P. (1957). On the role of croton oil in tumour formation. *Cancer Res.*, **17**, 71.

BOUTWELL, R.K., VERMA, A.K., ASHENDEL, L. & ASTRUP, E. (1982). Mouse skin: A useful model system for studying the mechanism of chemical carcinogenesis. In: *Carcinogenesis,* Vol. 7. "Co-carcinogenesis and Biological Effects of Tumour Promoters". (Eds. Hecker *et al.*), New York: Raven Press, p. 1.

BRALOW, S.P., GRUNSTEIN, M. & MERANZE, D.R. (1973). Host resistance to gastric adenocarcinomatosis in three strains of rats ingesting *N*-methyl-*N*-nitro-*N*-nitrosoguanidine. *Oncology,* **27,** 168.

COLEMAN, G.L., BARTHOLD, S.W., OSBALDISTON, G.W., FOSTER, S.J. & JONAS, A.M. (1977). Pathological changes during aging in barrier-reared Fischer 344 male rats. *J. Gerentol.,* **32,** 258.

FUSENIG, N.E. & DZARLIEVA, R.T. (1982). Phenotypic and chromosomal alterations in cell cultures as indicators of tumours-promoting activity. In: *Carcinogenesis,* Vol. 7. "Co-carcinogenesis and Biological Effects of Tumour Promoters. (Eds. Hecker *et al.*), New York: Raven Press, p. 201.

GOODMAN, D.G., WARD, J.M., SQUIRE, R.A., CHU, K.C. & LINHART, M.S. (1979). Neoplastic and nonneoplastic lesions in aging F344 rats. *Toxicol. Appl. Pharmacol.,* **48,** 237.

HICKS, R.M. (1983*a*). Pathological and biochemical aspects of tumour promotion. *Carcinogenesis,* **4,** 1209.

HICKS, R.M. (1983*b*). Multi-stage tumour development in the urinary bladder. In: *13th International Cancer Congress, Part B. Biology of Cancer* (1), New York: Alan R. Liss, Inc., p. 205.

HICKS, R.M., WAKEFIELD, J.St.J. & CHOWANIEC, J. (1975). Evaluation of a new model to detect carcinogens and co-carcinogens: results obtained with saccharin, cyclamate and cyclophosphamide. *Chem. Biol. Interact.,* **11,** 225.

HICKS, R.M., WRIGHT, R. & WAKEFIELD, J.St.J. (1982). The induction of rat bladder cancer by 2-naphthylamine. *Br. J. Cancer,* **46,** 646.

KINSELLA, A.R. & RADMAN, M. (1978). Tumour promoter induces sister chromatid exchanges: Relevance to mechanisms of carcinogenesis. *Proc. Natl Acad. Sci.,* **75,** 6149.

LAWLEY, P.D. (1976). Carcinogenesis by alkylating agents. In: *ACS Monograph* **173**. *Chemical Carcinogens,* (Ed. C.E. Searle), Washington D.C.: p. 83.

LAWLEY, P.D. (1980). DNA as a target of alkylating carcinogens. *Br. Med. Bull.,* **36,** 19.

LITTLEFIELD, N.A., GREENMAN, D.L., FARMER, J.H. & SHELDON, W.G. (1979). Effects of continuous exposure to 2-AAF on urinary bladder hyperplasia and neoplasia. *J. Environ. Pathol. Toxicol.,* **3,** 35.

MARTIN, M.S., MARTIN, F., JUSTRABO, E., MICHIELS, R., BASTEIN, H. & KNOBEL, S. (1974). Susceptibility of inbred rats to gastric and duodenal carcinomas induced by *N*-methyl-*N*-nitro-*N*-nitrosoguanidine. *J. Natl Cancer Inst.,* **53,** 837.

MOOLGAVKER, S.H. & KNUDSON, A.G. (1981). Mutation and cancer: A model for human carcinogenesis. *J. Natl Cancer Inst.,* **66,** 1037.

PERRY, P. & EVANS, H.J. (1975). Cytological detection of mutagen-carcinogen exposure by sister chromatid exchange. *Nature,* **258,** 121.

PETERSON, A.R., PETERSON, H. & HEIDELBERGER, C. (1979). Oncogenesis, mutagenesis, DNA damage and cytotoxicity in cultured mammalian cells treated with alkylating agents. *Cancer Res.,* **39,** 131.

ROBERTS, J.J., STURROCK, J.E. & WARD, K.N. (1974). The enhancement by caffeine of alkylation-induced cell death, mutations, and chromosomal abberations in Chinese hamster cells, as a result of inhibition of post-replication DNA repair. *Mutat. Res.,* **26,** 129.

ROE, F.J.C. (1956). The development of malignant tumours of mouse skin after "initiating" and "promoting" stimuli. III. The carcinogenic action of croton oil. *Br. J. Cancer,* **10,** 72.

SASS, B., RABSTEIN, L.S., MADISON, R., NIMS, R.M., PETERS, R.L. & KELLOFF, G.L. (1975). Incidence of spontaneous neoplasms in F344 rats throughout the natural life-span. *J. Natl Cancer Inst.,* **54,** 1449.

SCRIBNER, N.K. & SCRIBNER, J.D. (1980). Separation of initiating and promoting effects of the skin carcinogen 7-bromo-methylbenz(a)anthracene. *Carcinogenesis*, **1**, 97.

SEVERS, N.J., BARNES, S.H., WRIGHT, R. & HICKS, R.M. (1982). Induction of bladder cancer in rats by fractionated intravesicular doses of *N*-methyl-*N*-nitrosourea. *Br. J. Cancer*, **45**, 337.

SLAGA, T.J. (1983). Overview of tumour promotion in animals. *Environ. Health Perspect.*, **50**, 3.

TUDOR, R.J., SEVERS, N.J. & HICKS, R.M. (1983). The induction of urothelial hyperplasia by methyl methanesulphonate and ethyl methanesulphonate. *Br. J. Cancer*, **48**, 289.

WAKEFIELD, J.St.J. & HICKS, R.M. (1974). Erythrophagocytosis by the epithelial cells of the bladder. *J. Cell Sci.*, **15**, 555.

WEINSTEIN, I.B. (1983). Protein kinase, phospholipid and control of growth. *Nature*, **302**, 750.

WERNER, E.A. (1919). The constitution of the carbamides. IX. The interaction of nitrous acid and mono-substituted ureas. The preparation of diazomethane, diazoethane, diazo-n-butane and diazoisopentane. *J. Chem. Soc.*, **115**, 1093.